# The shutting down of the insulin pathway: a developmental window for *Wolbachia* load and feminization

**DOI:** 10.1038/s41598-020-67428-1

**Published:** 2020-06-29

**Authors:** Benjamin Herran, Sandrine Geniez, Carine Delaunay, Maryline Raimond, Jérôme Lesobre, Joanne Bertaux, Barton Slatko, Pierre Grève

**Affiliations:** 1 Laboratoire Ecologie et Biologie des Interactions - UMR CNRS 7267 - Equipe Ecologie, Evolution, Symbiose - Université de Poitiers, 5 rue Albert Turpain, TSA 51106, 86073 Poitiers Cedex 9, France; 2 New England Biolabs, Inc., 240 County Road, Ipswich, MA USA; 3 Present Address: Laboratoire Microorganismes: Génome et Environnement, UMR CNRS 6023, Université Clermont Auvergne, 63178 Aubière, France

**Keywords:** Developmental biology, Microbiology

## Abstract

Using the isopod *Armadillidium vulgare* as a case study, we review the significance of the "bacterial dosage model", which connects the expression of the extended phenotype to the rise of the *Wolbachia* load. In isopods, the Insulin-like Androgenic Gland hormone (IAG) induces male differentiation: *Wolbachia* feminizes males through insulin resistance, presumably through defunct insulin receptors. This should prevent an autocrine development of the androgenic glands so that females differentiate instead: feminization should translate as *IAG* silencing and increased *Wolbachia* load in the same developmental window. In line with the autocrine model, uninfected males expressed IAG from the first larval stage on, long before the androgenic gland primordia begin to differentiate, and exponentially throughout development. In contrast in infected males, expression fully stopped at stage 4 (juvenile), when male differentiation begins. This co-occurred with the only significant rise in the *Wolbachia* load throughout the life-stages. Concurrently, the raw expression of the bacterial Secretion Systems co-increased, but they were not over-expressed relative to the number of bacteria. The isopod model leads to formulate the "bacterial dosage model" throughout extended phenotypes as the conjunction between bacterial load as the mode of action, timing of multiplication (pre/post-zygotic), and site of action (soma vs. germen).

## Introduction

*Wolbachia* are likely the most widespread endosymbionts on Earth, infecting arthropods such as insects, mites, spiders and crustaceans, but also parasitic nematodes^1^. These maternally transmitted endosymbionts proliferate by manipulating the reproduction of their host through four main extended phenotypes (male-killing, parthenogenesis, Cytoplasmic Incompatibility (CI), feminization) or by developing an obligate interaction with their partner^2,3^. The expression of the extended phenotypes was repeatedly related to the bacterial load. In a seminal study on *Nasonia* sp. and CI, a “bacterial dosage model” was proposed in which a cross is effective when the oocyte harbours equal or greater numbers of *Wolbachia* than the spermatocytes^4^. Basically, in incompatible crosses, CI prevents normal mitosis at the first embryonic division or during embryogenesis, leading to embryonic mortality^5,6^. In *Nasonia* sp*.* however, the bidirectional CI is complicated by the haplodiploid determination system. Incompatible crosses result in the elimination of paternal chromosomes and therefore yield only haploid males, as if the eggs had not been fertilized^7^. In compatible crosses, antibiotic curing of *Wolbachia* in mothers gradually decreases the bacterial load in the oocytes, shifting the sex ratio in successive broods from all-female to all-male^4^. As incompatibility translates the non-rescue from a toxin secreted by the bacteria in the sperm, this “bacterial dosage model” reflects a pre-zygotic action of *Wolbachia* in male cysts. Similar inferences were further proposed from correlating CI levels and cyst infection frequency. In *Drosophila melanogaster*, *w*Mel infects only 8% of testes cysts and displays a low CI effect^8^. When transinfected in *D. simulans*, the CI effect shifts to high, while cyst infection reaches 80%^8^. Similar correlations were made in different *w*Ri-infected *Drosophila* hosts^9,10^. Furthermore, quantitative analyses in several species such as *D. simulans*, *Aedes albopictus* or the isopod *Porcellio dilatatus* also correlated the strength of unidirectional CI with the *Wolbachia* load^11,12^.

The “bacterial dosage model” can be expanded to the other extended phenotypes. In *D. bifasciata* harbouring a male-killing strain, high temperatures reduce the bacterial load in mothers to a threshold below which *Wolbachia* is no longer able to kill males, thus producing *Wolbachia*-bearing males^13^. In *Hypolimnas bolina*, preventing bacterial proliferation in mothers postpones the death of the sons to a larval stage, upon *Wolbachia* recovery^14^. In *Muscidifurax uniraptor*, the *Wolbachia*-induced production of diploid females through parthenogenesis is altered in a dose-dependent manner by rifampicin treatments of the mothers, that result in a decrease of the bacterial load: the higher the dose of rifampicin, the higher the proportion of haploid males^15^. Again, and in both cases, the proper execution of the extended phenotypes is conditioned by the bacterial load at a pre-zygotic level, as depleting the generation N-1 hampers their expression. As for effectors, there is no telling whether they are produced in the zygote, or stock-piled in the maternal generation, mirroring the paternal toxin in CI.

In contrast, mutualistic and feminizing strains mostly act at the post-zygotic level, continuously throughout the embryonic and/or larval development, or even throughout the life of their host. In the mutualistic relationship with *Brugia malayi*^16^, microfilariae and L1 to L3 larval stages in the mosquito host are poorly infected, whereas the *Wolbachia* load dramatically increases in L3 larvae upon transmission to a mammalian host^17^. According to Landmann et al.,^18^ antibiotic treatments disrupt especially L3/L4 larvae development, probably as a consequence of inhibited bacterial division^17^. These observations suggest that *Wolbachia* is involved in the development of late larval stages, possibly participating to a kind of metabolic complementation scheme^18^. *Wolbachia* similarly conditions embryo and adult survival, although this was not connected with a heightened bacterial load^18,19^. In the bed bug *Cimex lectularius*, *Wolbachia* provides the host with B vitamins, leading to a nutritional mutualism^20,21^. *Wolbachia*-cured embryos and larvae suffer growth defects and display a lower adult emergence rate. Bacterial titres increase dramatically between the first and the fifth instar stages, correlatively to the essential role of *Wolbachia* for embryonic development^22^.

Very much in contrast with the other reproductive extended phenotypes, feminization displays a diversity of mechanisms, well reflected in the comparison of three *Wolbachia* strains. All systems generate phenotypic functional females, but with different genotypic identities: “de-sexualized” Z0 (*Eurema mandarina*, known first as *E. hecabe* yellow-type), genetic females XX (*Zyginidia pullula*), genetic males ZZ (*Armadillidium vulgare*)^23–26^. If the prevalence of females increases, so does the transmission of *Wolbachia*: this is the case in *E. mandarina* (~ 100% females), *A. vulgare* (~ 80% females), but not in *Z. pullula* (~ 50% females). Indeed in *Z. pullula*, infected genetic males (X0) become intersexes with more or less damaged ovaries: they are mostly dead-ends as progenies are very seldom observed^26,27^. Here, feminization swaps the sex-specific profile of the genomic imprinting patterns from male to female, ahead of sex realisation cascades and differentiation processes, possibly targeting a master control of the latter^28^. That a threshold load of bacteria is necessary for feminization is expected from the observation that 1% intersexes retain testes instead of ovaries, and a male imprinting profile instead of a female one, in conjunction with a lower bacterial load^28,29^. In contrast, feminization in *E. mandarina* acts as a two-step mechanism, during sex determination and differentiation. *Wolbachia*-infected females are Z0 instead of WZ, following the exclusion of the maternal sex chromosomes during or after meiosis, so that each new generation is Z0^24,30^. Whereas *Wolbachia*-cured Z0 individuals are not viable, in infected ones *Wolbachia* compensates for the absence of the W chromosome somewhere along the sex realisation cascade, ultimately imposing the female *doublesex* splicing variant^24^. That the male splicing variant could or should be expressed instead is revealed by curing individuals during the larval development, which results in the differentiation of intersexes expressing both splicing variants, in conjunction with decreased bacterial loads^24^.

In our model *A. vulgare*, feminization results in sex reversal and appears to be purely post-zygotic. Here, sex differentiation is orchestrated by a masculinising hormone produced by the androgenic glands: the Insulin-like Androgenic Gland hormone (IAG)^31,32^. It supersedes the sex-determination processes. Indeed, sex differentiation at the juvenile stage 4 can be surgically reversed for yet another couple of moults: WZ females are fully masculinised by the grafting of an androgenic gland^33^, while ZZ males spontaneously become fertile females upon the ablation of the primordia of the androgenic glands^34^. Therefore, female is the default sex. In ZZ males, the androgenic gland primordia begin to proliferate at stage 4^35^ and become fully visible as tissues at stage 6^36^. *Wolbachia* infection shunts this process, without altering the number of chromosomes^37^: ~ 80% of a progeny turn into functional phenotypic females and occasional intersexes, while the remaining ~ 20% males result from incomplete *Wolbachia* transmission^38^. In infected, phenotypic females, the androgenic glands never become visible, but can be revealed upon partial curing by temperature: in each gonad, the third androgenic gland primordium re-activates, leading to re-masculinisation^39^. This shows that they retain a functional male determination and differentiation pathway, and that it is the latter that would be dampened somehow by *Wolbachia*. In intersexes, feminization is delayed, so that female-like intersexes retain a single vestigial androgenic gland per gonad, while male-like intersexes display fully developed and even hypertrophied androgenic glands; however, if cut, their copulatory pleopods regenerate in a female form^25^. In fact, all infected individuals become impervious to the masculinising effect of the IAG, be it synthesized by their own glands or by grafted ones: in other words, feminization is a form of insulin resistance. According to Juchault and Legrand^40^, it is the receptor of the IAG (the insulin-like hormone) that would be defunct. This hints to an autocrine mechanism in the differentiation of the androgenic glands, where shunting these receptors would prevent their development. Hence, the question that arises is when does IAG refractoriness occur and is it related to increased *Wolbachia* densities?

Here, we report that genetic males expressed the IAG during the larval stages even before the androgenic gland primordia begin to differentiate, and in accordance with their ZZ genotype, regardless of the presence or absence of *Wolbachia*. However, in infected individuals, the *IAG* gene expression fully stopped at the juvenile stage 4, 5 or 6 at the latest, i.e. the time-window for male differentiation. This was matched by a bacterial-load increase at stage 4. Together with the *Wolbachia* depletion experiments of Rigaud et al.^41^, our results show that, in this model again, the bacterial load is instrumental to the execution of the extended phenotype. Concurrently, the raw expression of the bacterial Secretion Systems (T1SS and T4SS) co-increased at stage 4, but they were not over-expressed relative to the number of bacteria. We discuss the possibility that effectors themselves could be constitutively expressed. The execution of any extended phenotype could therefore reflect the conjunction of bacterial load, timing of multiplication, and site of multiplication or invasion, adding further dimensions to the bacterial dosage model.

## Results and discussion

**Figure 1:**
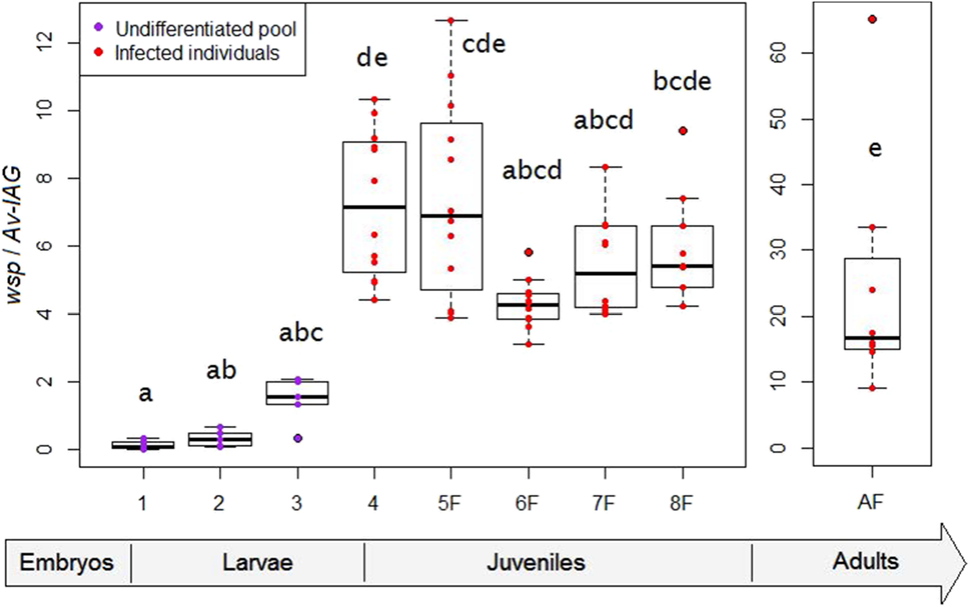
Evolution of the *Wolbachia* load during development in the *Wolbachia*-infected lineage of *A. vulgare*. The number of bacteria was estimated by qPCR using the *wsp* gene, normalised by the single copy nuclear gene *Av-IAG* from the host. Pools of undifferentiated larvae were sampled after birth (stage 1), one and two weeks after birth (stages 2 and 3, respectively). For the next developmental steps, *Wolbachia*-infected animals were sampled individually: undifferentiated juveniles (stage 4), and phenotypic females (stages 5–8) until adulthood (AF for adult females).

To compare the infection load of animals of different developmental stages (Fig. 1), we quantified the number of *Wolbachia* genomes relative to the number of host cells using the ratio of two single-copy genes: the *wsp* gene for *Wolbachia* and the *IAG* gene for the host^42^. The bacterial load was quite constant during the larval stages (1–3; *p *values > 0.05), during the juvenile stages and in young adults (stages 4–8; *p* values > 0.05). In between, the bacterial load rose sharply (4.9-fold between stages 3 and 4; *p* value = 0.027). In comparison with stage 8, the 1-year-old adults displayed a 4-fold increase, which was however not significant (*p* value = 0.208), with a high variation between individuals. Here, inter-individual variability was likely inflated by fluctuations of the *Wolbachia* load during the reproductive cycle, for example when *Wolbachia* accumulates in the ovaries: more oocytes get infected as ovaries mature^38^. Overall, the only effective increase in the bacterial load coincided with the time of sexual differentiation: in isopods, the gonads start to differentiate at stage 4, then at stage 5 the external sex characters begin to appear in males^36^.

**Figure 2:**
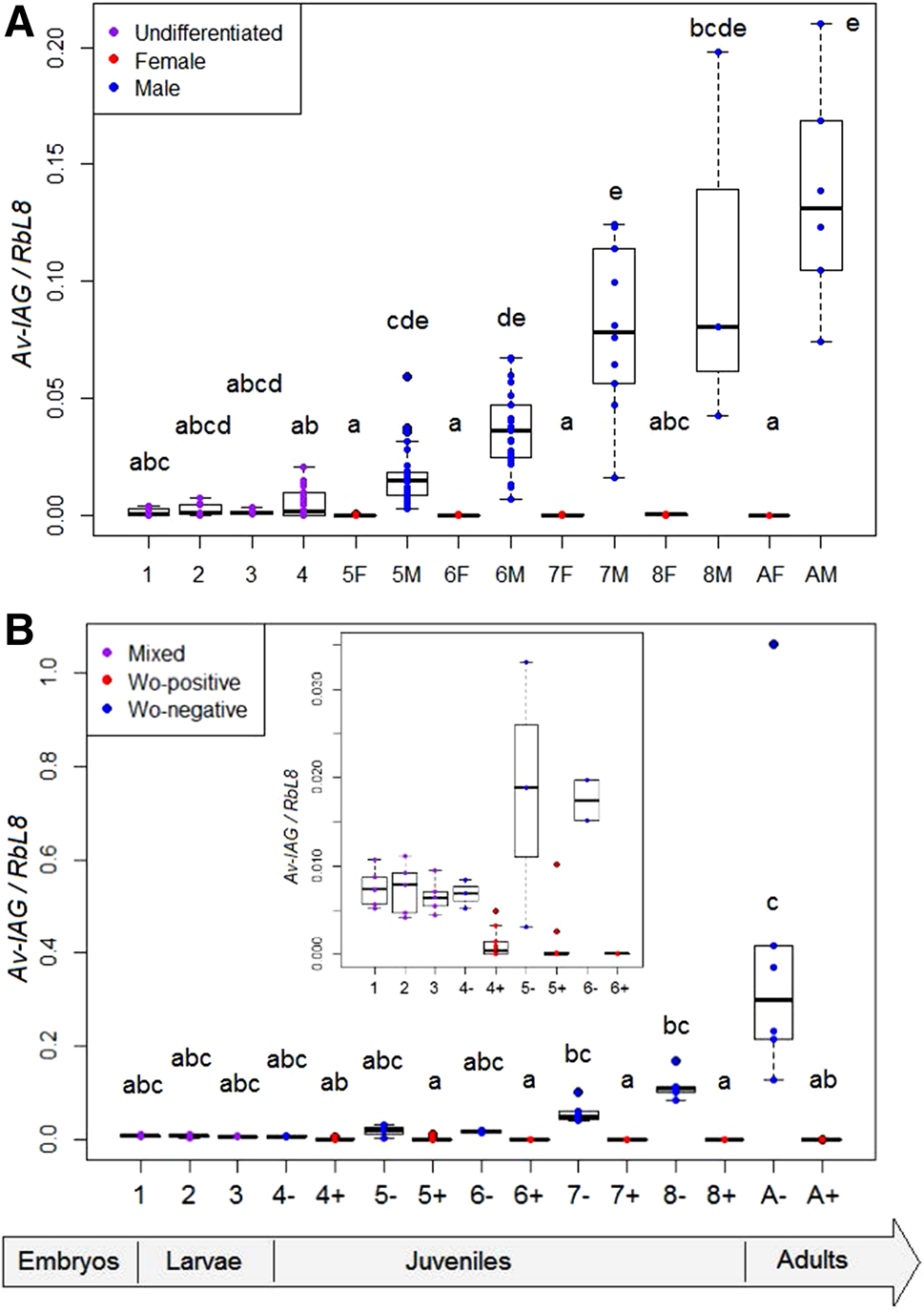
RT-qPCR expression profiles of the *Av-IAG* mRNA during development in uninfected (**A**) and *Wolbachia*-infected lineages (**B**). The expression level of the *Av-IAG* gene was normalised to the one of the *RbL8* housekeeping gene. Pools of undifferentiated larvae were sampled after birth (stage 1), one and two weeks after birth (stages 2 and 3, respectively). Animals were sampled individually for stage 4 (undifferentiated juveniles), stages 5–8 and into adulthood: (**A**) genetic males and females (AM for adult males, AF for adult females) or (**B**) *Wolbachia*-infected or uninfected individuals (A+, A−); insert: focus on stages 1–6.

In *A. vulgare*, feminization is expected to disrupt the autocrine development of the androgenic glands: it implies that in normal males, the IAG should be expressed early in their precursor cells, even before the glands differentiate at stage 6^36^. Precursor cells were described by Juchault^35^ at stages 4 and 5, when male gonads differentiate, in the suspensory tracts where the glands will develop. He also predicted that the precursors already exist in the undifferentiated gonads in the larval stages. Congruently, in the males of the *Wolbachia*-free lineage, the *IAG* gene was expressed from birth on and exponentially throughout development, including in the larval stages (1–3) and in differentiating juveniles (4–6) (Fig. 2A). Females on the other hand are not expected to express the *IAG* gene: this was however reported in some decapod models, in connection with a supplementary role in metabolism (e.g.^43^). Since we sampled the larval stages as pools that contained genetic females as well, we cannot verify whether larval females expressed the IAG initially. But even if they did, and at a significant rate, it would not matter for differentiation: Suzuki^44^ demonstrated that larval females are refractory to IAG and cannot be reversed by the graft of an androgenic gland. As concerns juvenile females, they did express the *IAG* gene (31/36 females from stages 5 to 8), but at an extremely low and constant rate (2.10^–4^ as a mean), that contrasted with the exponential expression in males. This resulted in an increasing gap between females and males, from a 79-fold difference at stage 5 to a 488-fold difference at stage 8 (Fig. 2A). In any case, such a weak expression was not sufficient to trigger a biological response in male differentiation. Overall, in line with the literature, we infer that the crux of male differentiation relates to the expression level of the *IAG* gene from stage 4 to stage 6, allowing the differentiation of the gonads and the androgenic glands.

In the *Wolbachia*-infected lineage, it is in this developmental window that we observed an endocrine disruption. The *IAG* gene was initially expressed in the larval pools, but at a rate that matched that of stage 4 uninfected males (Fig. 2B), and that was even higher than in the uninfected lineage where the prevalence of males is 50% (Mann–Whitney test; stage 1: mean = 0.006 vs. 0.001, *p* value = 0.028; stage 2: mean = 0.006 vs. 0.004, *p* value = 0.18, ns; stage 3: mean = 0.005 vs. 0.001, *p* value = 0.002). Therefore, the larvae seemed to express the *IAG* gene according to their genotype (100% genetic males), not their future phenotype which depends on the *Wolbachia* infection status (~ 20% future males lacking *Wolbachia*, ~ 80% future females with *Wolbachia*). This expression of the *IAG* gene in infected animals was however in conjunction with a low *Wolbachia* load in larvae (Fig. 1). In contrast, from stage 4 on, the expression of the *IAG* mRNA was related to the *Wolbachia* infection status (Fig. 2B). Uninfected juveniles all expressed the *IAG* mRNA, whereas most *Wolbachia*-positive juveniles did not, despite their ZZ genotype. Some juveniles expressed the *IAG* gene while being infected by *Wolbachia*, but their prevalence dropped to zero over two stages: from 31% (5/16) at stage 4, 14% (2/14) at stage 5, to none in the later stages (stage 6: 0/11; stage 7: 0/18; stage 8: 0/14). They can correspond to future phenotypic females, *Wolbachia*-infected individuals being insensitive to IAG^40^, or to intersexes. However, the prevalence of intersexes in this lineage is much lower than this: over the last seven years only 10 intersexes were harvested among 2,532 individuals (0.39%) of 69 litters from our rearing. These individuals were thus probably future functional females wherein the *IAG* gene expression was about to get silenced in the presence of *Wolbachia*.

In either case, the time-window for a successful feminization in *A. vulgare* can be narrowed down to stages 4 and 5, which co-occurred with the only significant rise in the *Wolbachia* load. Earlier, even regular females are refractory to IAG^44^, so that *Wolbachia* preventing the expression of the IAG would be superfluous. Later, once the precursors of the androgenic glands degenerate (during stage 6^39^), females are terminally differentiated, so that they do not produce IAG anyway. In the fertile, female-like intersexes, male-differentiation is probably shunted in this window as well: only the first pair of androgenic gland primordia begins to develop and aborts. In contrast, in the sterile, male-like intersexes, feminization is delayed beyond these stages so that they have time to develop three functional pairs of androgenic glands before they become refractory to IAG^40^. Imperfect feminization could result from an insufficient *Wolbachia* load, leading to a belated extinction of the *IAG* gene, allowing partial male differentiation. Indeed, partial curing by temperature during larval development generates an excess of male-like intersexes^41^. Moreover, Rigaud et al.^45^ inferred from bioassays that female-like intersexes contain less bacteria than phenotypic females in their somatic tissues, whereas the bacterial load in ovaries is similar. While infecting ovaries is important for vertical transmission, disabling the receptors of the IAG for feminization is a body-wide symptom. A question is whether the heightened *Wolbachia* load serves to match such a broad target, or, closer to the model of Juchault and Legrand^40^, to reach and disable discrete endocrine centres that control the functionality of all IAG receptors^46^. In other words, within the “bacterial dosage model”, we consider the localisation of the heightened dose of *Wolbachia* in terms of site of action for the extended phenotype.

In this, *Wolbachia* strains that act at the post-zygotic level share common traits that contrast with those acting at the pre-zygotic level. For the latter, the site of expression of the extended phenotype matches the site of vertical transmission: it is a common target, to be reached in the N-1 generation. That location matters could therefore not be a part of the initial model of Breeuwer and Werren in CI^4^. The post-zygotic acting *Wolbachia* on the other hand, must target a somatic niche in addition to the germinal niche, the expression of the extended phenotype in the soma promoting indirectly vertical transmission in the germen. Similar to what we observed in *A. vulgare*, the execution of the extended phenotype is connected to a heightened bacterial load in the developmental stages. In *B. malayi*, this increase is observed in the somatic lateral chords where *Wolbachia* would complement host metabolism, even before the ovaries get infected in L4 females^47,48^. In *C. lectularius*^20,22^, *Wolbachia* is concentrated in the bacteriome plus ovaries: the increase of the bacterial load in individuals along larval development probably reflects the expansion of *Wolbachia* in these niches, following an early colonization in embryogenesis. Slightly closer to our model, *E. mandarina* harbours a feminizing *Wolbachia* strain; still, the differences with the isopod system are of a magnitude, since sex determination in insects is "cell-autonomous" and is enforced by differentiation, cell by cell. In the absence of a hormonal coordination, *Wolbachia* needs to act earlier than the larval stage, during embryogenesis, so that a homogenous sex background emerges in each cell^49^. In this, *Wolbachia* may need to be represented in all cells. Its action (possibly its global colonization) must be sustained further, all along development, or intersexes are obtained. Intersexes express both the male and the female splicing variants of *doublesex*, and are mosaics of sexual characters^24,49^: maybe it is a question of bacterial load at the scale of the cells, that would result in a mosaic expression of the splicing variants. In *A. vulgare*, we rather suspect that *Wolbachia* targets specific endocrine cells.

**Figure 3:**
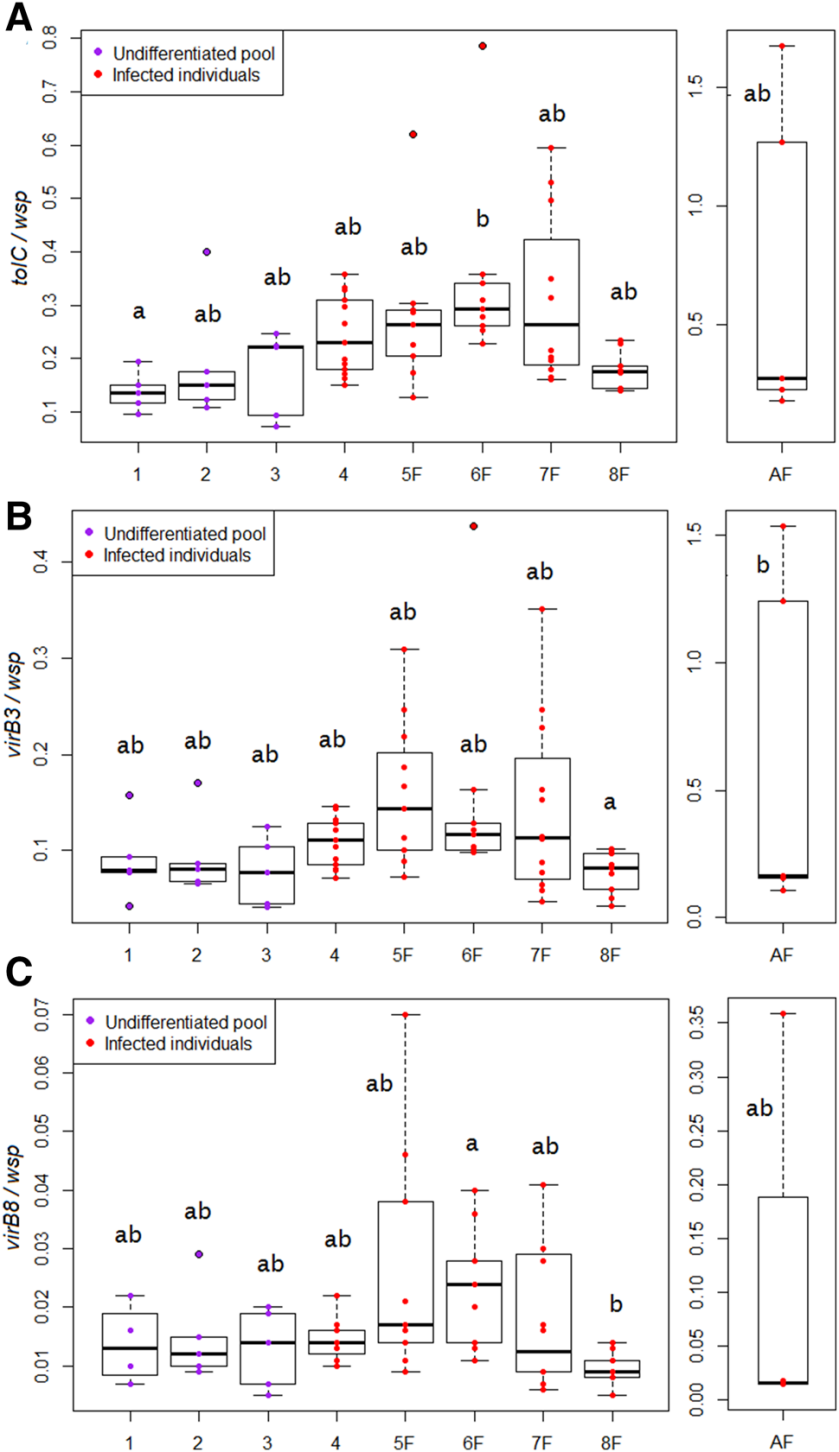
RT-qPCR expression profiles of the T1SS and T4SS genes during development in the *Wolbachia*-infected lineage of *A. vulgare*. The expression level of the *tolC* (**A**), *virB3* (**B**) and *virB8* (**C**) genes were normalised to the one of the *wsp* transcripts. Pools of undifferentiated larvae were sampled after birth (stage 1), one and two weeks after birth (stages 2 and 3, respectively). For the next developmental steps, *Wolbachia*-infected animals were sampled individually: undifferentiated juveniles (stage 4), and phenotypic females (stages 5–8) until adulthood (AF for adult females).

The “bacterial dosage model” entails a further dimension: the mode of action of *Wolbachia* to execute the extended phenotype. Intrinsic to this concept is that it is mediated through, so to speak, strength in numbers. Mechanistically, this should translate as an increased delivery of bacterial substances in the host cytosol, namely metabolites or effectors, transferred through secretion systems^50^. Stage-specific expressions or over-expressions could complement this process, but this is not quite the picture drawn in the literature. So far, only putative effectors are known, except for CI, *cifA* and *cifB* causing mitotic failures in the first stages of embryonic development^6,51^. They are expressed in succession within the window of heightened *Wolbachia* load in sperm cysts, and *cifA*, which doubles as a rescuer, in adult females^9,52^. They are however also expressed throughout embryonic development, by the bacteria inherited from the mother^52^. *cifA* itself is expressed only at very low levels in the early stages: rescue could stem from stock-piled maternal CifA instead, mirroring the paternal origin of the toxin. As regards post-zygotic acting *Wolbachia*, global transcriptomic studies in filarial models record L3/L4-specific products^53^, differentially expressed products^54^ or enriched GO-terms^55^, but these data are not normalized against the *Wolbachia* load. As for secretion systems, T4SS is expressed continuously throughout the life stages^56,57^. Normalizing its expression or that of its transcription factors (*w*BmxR1 and *w*BmxR2) against the *Wolbachia* load reveals an under-expression only in microfilariae^57^. In *A. vulgare*, T1SS and T4SS were identified in the ongoing sequencing project of *w*VulC (Liu et al*.*, unpublished results). The T1SS is encoded by three genes (*tolC*, *hlyB*, *hlyD*) scattered in the genome, and the T4SS by the *virB3-virB6* and the *virB8-virD4* operons. Hence, we followed the expression of these secretion systems through that of the *tolC*, *virB3* and *virB8* genes along the development stages of *A. vulgare*. While the raw expression of the three genes increased from stage 4 on, the normalized expression remained constant from stage 1 to stage 8, and in adult females (Fig. 3A–C). Like the bacterial load, the gene expression was highly variable in adults, likely due to the different reproductive states of the females^38^. The permanent expression of the genes encoding these secretion systems in *A. vulgare* and in nematode models may result from their involvement in symbiotic processes that go beyond the execution of the extended phenotype. All the same, for these systems and for effectors, a regulation of expression through bacterial numbers may prove more plastic and adaptive than a regulation wired in transcription dynamics, that demands synchronising with pinpoint accuracy not only with the host's cell, but with its life cycle.

## Conclusion

In unifying our perception of the "bacterial dosage model" throughout the extended phenotypes of *Wolbachia*, we have formulated this concept through the notions of timing of expression (pre/post-zygotic acting strains), site of action, and mode of action. In *A. vulgare*, analysing in situ the fine distribution of *Wolbachia* within the tissues during the different stages of development will allow to discriminate the site of action of feminization from the site of vertical transmission. It will determine whether site colonization stems from distributing bacteria between daughter cells during embryogenesis, or entails a posterior migration of bacteria between organs. Indeed, in *B. malayi*, the larval gonads are free of *Wolbachia* until they are secondarily recolonised by the bacteria from the lateral chords^47,48^. At the interface between site and mode of action, Juchault and Legrand^40^ predict that *Wolbachia* invades the endocrine centres that control the activity of the receptors of the IAG (i.e. insulin receptors): grafting tissues containing these centres to male-like intersexes restores insulin sensitivity and therefore male differentiation. In other words, through *Wolbachia*, we are in search of an unforeseen level of control in the insulin pathway: a canonical switch in insulin sensitivity, the shutting of which leads to insulin resistance.

## Material and methods

### Biological material

*Armadillidium* *vulgare* (Malacostraca, Isopoda) individuals used in this study come from two lineages: a *Wolbachia*-free lineage originating from Nice (France) and a *Wolbachia*-infected lineage originating from Celles-sur-Belle (France), wherein females harbour the feminizing *w*VulC strain^58^. These lineages have been stably maintained in the laboratory since 1967 and 1991, respectively. All animals were reared under laboratory conditions in boxes containing wet compost and food ad libitum (dried lime tree leaves and fresh slices of carrots), at 20 °C, under the natural photoperiod.

In both lineages, several crosses were followed up in order to harvest animals at each post-embryonic developmental stage^36^. Larval stages were sampled in pools of 20–40 newly hatched individuals for stage 1, pools of 10–20 individuals one week later for stage 2 and two weeks after birth for stage 3. Juvenile animals were sampled individually and corresponding stages (4–8) were determined according to their size^36^. Finally, one-year-old males and females were sampled, well after sexual differentiation. All samples were stored immediately after harvesting in liquid nitrogen. The experiment was performed on at least 5–6 biological replicates for pooled samples, 9–13 individual juveniles and 5–6 biological replicates per adult.

### RNA and DNA extraction

Whole animals or animal pools were homogenized using a Vibra Cell 75,185 sonicator (amplitude of 35%). RNA and DNA were extracted from each *A. vulgare* sample using the Qiagen AllPrep DNA/RNA Mini Kit according to the manufacturer’s recommendations. RNA and DNA quantity was assessed by NanoDrop spectrophotometry.

### Quantification of the *Wolbachia* load by quantitative PCR (qPCR)

*Wolbachia* density was determined in each DNA sample previously extracted from young *A. vulgare* at different post-embryonic stages by qPCR amplification of the *Wolbachia* surface protein (*wsp*) gene from *Wolbachia* and the *IAG* gene from *A. vulgare*.

The qPCR reactions were performed using Applied Biosystems SYBR Green master mixes (5 μL Sybergreen 5X, 0.5 μL of each primer (10 µM; Supplementary Table S1), about 20 ng of DNA and sterile water up to 10 μL). The reactions were performed with a technical replicate on a LightCycler 480 System (Roche), using the following program: 95 °C for 10 min followed by 45 cycles of (95 °C for 10 s, 60 °C for 10 s, 72 °C for 20 s). Melting curves were established (65–97 °C) to check the specificity of the PCR products. The bacterial density was calculated in copy number of the bacterial genome normalized to the copy number of the host genome using Ct values and the LightCycler 480 Software. Statistically significant differences between groups were analysed with a Kruskal test followed by Dunn’s post-hoc tests implemented in the R package PMCMR^59^ using a *p *value = 0.05 and the Holm’s correction for multiple comparisons. The differential expression of the *IAG* gene between the *Wolbachia*-infected and the *Wolbachia*-free lineages was checked with a Mann–Whitney test, as specified in the text.

### Quantification of gene expression by reverse transcription (RT)-qPCR

The expression of the *IAG* gene was evaluated during *A. vulgare* development. T1SS and T4SS expression were also measured by quantification of the expression of *tolC*, *virB3* and *virB8* genes, using RNA of the same samples for which the bacterial load has been determined. RT were carried out on 500 ng of total RNA using random primers and the SuperScript III Reverse Transcriptase (Thermo Fisher Scientific) following the supplier's instructions. qPCR reactions were performed as already described using 2.5 μL cDNA and primers given in Supplementary Table S1. The *IAG* and secretion system gene expression levels were analysed relatively to the *RbL8* and *wsp* gene expression, respectively, using Ct values and the LightCycler 480 Software. Statistically significant differences between groups were analysed as described above.

## Supplementary information

Supplementary Table S1

## References

[1] Zug R, Hammerstein P (2012). Still a host of hosts for *Wolbachia*: analysis of recent data suggests that 40% of terrestrial arthropod species are infected. PLoS ONE.

[2] Landmann F (2019). The *Wolbachia* endosymbionts. Microbiol. Spectr..

[3] Taylor MJ, Voronin D, Johnston KL, Ford L (2013). *Wolbachia* filarial interactions. Cell. Microbiol..

[4] Breeuwer JA, Werren JH (1993). Cytoplasmic incompatibility and bacterial density in *Nasonia vitripennis*. Genetics.

[5] Serbus LR, Casper-Lindley C, Landmann F, Sullivan W (2008). The genetics and cell biology of *Wolbachia*-host interactions. Annu. Rev. Genet..

[6] LePage DP (2017). Prophage WO genes recapitulate and enhance *Wolbachia*-induced cytoplasmic incompatibility. Nature.

[7] Breeuwer JA, Werren JH (1990). Microorganisms associated with chromosome destruction and reproductive isolation between two insect species. Nature.

[8] Poinsot D, Bourtzis K, Markakis G, Savakis C, Merçot H (1998). *Wolbachia* transfer from *Drosophila melanogaster* into *D. simulans*: host effect and cytoplasmic incompatibility relationships. Genetics.

[9] Clark ME, Veneti Z, Bourtzis K, Karr TL (2002). The distribution and proliferation of the intracellular bacteria *Wolbachia* during spermatogenesis in *Drosophila*. Mech. Dev..

[10] Veneti Z (2003). Cytoplasmic incompatibility and sperm cyst infection in different *Drosophila*-*Wolbachia* associations. Genetics.

[11] Sinkins SP, Braig HR, O’Neill SL (1995). *Wolbachia pipientis*: bacterial density and unidirectional cytoplasmic incompatibility between infected populations of *Aedes albopictus*. Exp. Parasitol..

[12] Sicard M (2014). Bidirectional cytoplasmic incompatibility caused by *Wolbachia* in the terrestrial isopod *Porcellio dilatatus*. J. Invertebr. Pathol..

[13] Hurst GD, Johnson AP, Schulenburg JH, Fuyama Y (2000). Male-killing *Wolbachia* in *Drosophila*: a temperature-sensitive trait with a threshold bacterial density. Genetics.

[14] Charlat S, Davies N, Roderick GK, Hurst GDD (2007). Disrupting the timing of *Wolbachia*-induced male-killing. Biol. Lett..

[15] Zchori-Fein E, Gottlieb Y, Coll M (2000). *Wolbachia* density and host fitness components in *Muscidifurax uniraptor* (Hymenoptera: pteromalidae). J. Invertebr. Pathol..

[16] Taylor MJ, Bandi C, Hoerauf A (2005). *Wolbachia*. Bacterial endosymbionts of filarial nematodes. Adv. Parasitol..

[17] McGarry HF, Egerton GL, Taylor MJ (2004). Population dynamics of *Wolbachia* bacterial endosymbionts in *Brugia malayi*. Mol. Biochem. Parasitol..

[18] Landmann F, Voronin D, Sullivan W, Taylor MJ (2011). Anti-filarial activity of antibiotic therapy is due to extensive apoptosis after *Wolbachia* depletion from filarial nematodes. PLoS Pathog..

[19] Wu B (2009). The heme biosynthetic pathway of the obligate *Wolbachia* endosymbiont of *Brugia malayi* as a potential anti-filarial drug target. PLoS Negl. Trop. Dis..

[20] Hosokawa T, Koga R, Kikuchi Y, Meng X-Y, Fukatsu T (2010). *Wolbachia* as a bacteriocyte-associated nutritional mutualist. Proc. Natl. Acad. Sci..

[21] Nikoh N (2014). Evolutionary origin of insect-*Wolbachia* nutritional mutualism. Proc. Natl. Acad. Sci. USA.

[22] Fisher ML (2018). Growth kinetics of endosymbiont *Wolbachia* in the common bed bug, *Cimex lectularius*. Sci. Rep..

[23] Hiroki M, Kato Y, Kamito T, Miura K (2002). Feminization of genetic males by a symbiotic bacterium in a butterfly, *Eurema hecabe* (Lepidoptera: Pieridae). Naturwissenschaften.

[24] Kageyama D (2017). Feminizing *Wolbachia* endosymbiont disrupts maternal sex chromosome inheritance in a butterfly species. Evol. Lett..

[25] Legrand JJ, Juchault P (1986). Rôle des bactéries symbiotiques dans l’intersexualité, la monogénie et la spéciation chez des crustacés oniscoïdes. Bolletino Zool..

[26] Negri I, Pellecchia M, Mazzoglio PJ, Patetta A, Alma A (2006). Feminizing *Wolbachia* in *Zyginidia pullula* (Insecta, Hemiptera), a leafhopper with an XX/X0 sex-determination system. Proc. R. Soc. B Biol. Sci..

[27] Negri I, Franchini A, Mandrioli M, Mazzoglio PJ, Alma A (2008). The gonads of *Zyginidia pullula* males feminized by *Wolbachia pipientis*. Bull. Insectol..

[28] Negri I (2009). Unravelling the *Wolbachia* evolutionary role: the reprogramming of the host genomic imprinting. Proc. R. Soc. B Biol. Sci..

[29] Negri I, Mazzoglio PJ, Franchini A, Mandrioli M, Alma A (2009). Male or female? The epigenetic conflict between a feminizing bacterium and its insect host. Commun. Integr. Biol..

[30] Kern P, Cook JM, Kageyama D, Riegler M (2015). Double trouble: combined action of meiotic drive and *Wolbachia* feminization in *Eurema* butterflies. Biol. Lett..

[31] Ventura T, Rosen O, Sagi A (2011). From the discovery of the crustacean androgenic gland to the insulin-like hormone in six decades. Gen. Comp. Endocrinol..

[32] Cerveau N, Bouchon D, Bergès T, Grève P (2014). Molecular evolution of the androgenic hormone in terrestrial isopods. Gene.

[33] Suzuki S, Yamasaki K (1997). Sexual bipotentiality of developing ovaries in the terrestrial isopod *Armadillidium vulgare* (Malacostraca, Crustacea). Gen. Comp. Endocrinol..

[34] Suzuki S, Yamasaki K (1991). Sex-reversal of male *Armadillidium vulgare* (Isopoda, Malacostraca, Crustacea) following andrectomy and partial gonadectomy. Gen. Comp. Endocrinol..

[35] Juchault, P. Contribution à l’étude de la différenciation sexuelle mâle chez les crustacés isopodes (Université de Poitiers, 1966). 4387384

[36] Suzuki S, Yamasaki K (1995). Morphological studies on sexual differentiation in *Armadillidium vulgare* (Isopoda: Armadillidae): androgenic gland and male sexual characters. Crustac. Res..

[37] Artault, J.-C. Contribution à l’étude des garnitures chromosomiques chez quelques crustacés isopodes (Université de Poitiers, 1977).

[38] Genty L-M, Bouchon D, Raimond M, Bertaux J (2014). *Wolbachia* infect ovaries in the course of their maturation: last minute passengers and priority travellers?. PLoS ONE.

[39] Juchault P, Martin G, Legrand JJ (1980). Induction par la température d’une physiologie mâle chez les néo-femelles et les intersexués du crustacé oniscoïde *Armadillidium vulgare* Latr., hébergeant un bactéroïde à action féminisante. Int. J. Invertebr. Reprod..

[40] Juchault P, Legrand JJ (1985). Contribution à l’étude du mécanisme de l’état réfractaire à l’hormone androgène chez les *Armadillidium vulgare* Latr. (crustacé, isopode, oniscoïde) hébergeant une bactérie féminisante. Gen. Comp. Endocrinol..

[41] Rigaud T, Juchault P, Mocquard JP (1991). Experimental study of temperature effects on the sex ratio of broods in terrestrial Crustacea *Armadillidium vulgare* Latr. Possible implications in natural populations. J. Evol. Biol..

[42] Dittmer J (2014). Host tissues as microhabitats for *Wolbachia* and quantitative insights into the bacterial community in terrestrial isopods. Mol. Ecol..

[43] Li S, Li F, Sun Z, Xiang J (2012). Two spliced variants of insulin-like androgenic gland hormone gene in the Chinese shrimp *Fenneropenaeus chinensis*. Gen. Comp. Endocrinol..

[44] Suzuki S (1999). Androgenic gland hormone is a sex-reversing factor but cannot be a sex-determining factor in the female crustacean isopods *Armadillidium vulgare*. Gen. Comp. Endocrinol..

[45] Rigaud T, Souty-Grosset C, Raimond R, Mocquard JP, Juchault P (1991). Feminizing endocytobiosis in the terrestrial crustacean *Armadillidium vulgare* Latr. (Isopoda): recent acquisitions. Endocyto Cell Res..

[46] Herran B, Bertaux J, Grève P (2018). Divergent evolution and clade-specific duplications of the Insulin-like receptor in malacostracan crustaceans. Gen. Comp. Endocrinol..

[47] Fischer K, Beatty WL, Jiang D, Weil GJ, Fischer PU (2011). Tissue and stage-specific distribution of *Wolbachia* in *Brugia malayi*. PLoS Negl. Trop. Dis..

[48] Landmann F (2012). Both asymmetric mitotic segregation and cell-to-cell invasion are required for stable germline transmission of *Wolbachia* in filarial nematodes. Biol. Open.

[49] Narita S, Kageyama D, Nomura M, Fukatsu T (2007). Unexpected mechanism of symbiont-induced reversal of insect sex: feminizing *Wolbachia* continuously acts on the butterfly *Eurema hecabe* during larval development. Appl. Environ. Microbiol..

[50] Bhattacharya T, Newton ILG (2019). Mi Casa es Su Casa: how an intracellular symbiont manipulates host biology. Environ. Microbiol..

[51] Beckmann JF, Ronau JA, Hochstrasser M (2017). A *Wolbachia* deubiquitylating enzyme induces cytoplasmic incompatibility. Nat. Microbiol..

[52] Lindsey ARI (2018). Evolutionary genetics of cytoplasmic incompatibility genes cifA and cifB in prophage WO of *Wolbachia*. Genome Biol. Evol..

[53] Bennuru S (2011). Stage-specific proteomic expression patterns of the human filarial parasite *Brugia malayi* and its endosymbiont *Wolbachia*. Proc. Natl. Acad. Sci..

[54] Luck AN (2014). Concurrent transcriptional profiling of *Dirofilaria immitis* and its *Wolbachia* endosymbiont throughout the nematode life cycle reveals coordinated gene expression. BMC Genom.s.

[55] Bennuru S (2016). Stage-specific transcriptome and proteome analyses of the filarial parasite *Onchocerca volvulus* and its *Wolbachia* endosymbiont. MBio.

[56] Grote A (2017). Defining *Brugia malayi* and *Wolbachia* symbiosis by stage-specific dual RNA-seq. PLoS Negl. Trop. Dis..

[57] Li Z, Carlow CK (2012). Characterization of transcription factors that regulate the type IV secretion system and riboflavin biosynthesis in *Wolbachia* of *Brugia malayi*. PLoS ONE.

[58] Bouchon D, Rigaud T, Juchault P (1998). Evidence for widespread *Wolbachia* infection in isopod crustaceans: molecular identification and host feminization. Proc. R. Soc. Lond. B Biol. Sci..

[59] Pohlert, T. The Pairwise Multiple Comparison of Mean Ranks Package (PMCMR) (2014).

