# Supplementary material for: The shutting down of the insulin pathway: a developmental window for Wolbachia load and feminization
Source: Sci Rep. 2020 Jun 29;10:10551. doi: 10.1038/s41598-020-67428-1 (PMC7324399; doi:10.1038/s41598-020-67428-1)
Supplement: Supplementary file 1 — Supplementary Table S1 [file 41598_2020_67428_MOESM1_ESM.pdf]

The shutting down of the insulin pathway: a developmental window for *Wolbachia* load and feminization.

Benjamin Herran, Sandrine Geniez, Carine Delaunay, Maryline Raimond, Jérôme Lesobre, Joanne Bertaux, Barton Slatko, Pierre Grève

Experiment	Name	Sequence	Size	Tm (°C)
qPCR	qWsp_F	TGGTGCAGCATTTACTCCAG	20	60,7
	qWsp_R	TCGCTTGATAAGCAAAACCA	20	57,7
	qAv-IAG_F	GAGGTATGAGATCCGATGTG	20	59.5
	qAv- IAG_R	AAATGCCAATTCATCTTCAGG	21	62.7
RT-qPCR	qAv-IAG_F	GAGGTATGAGATCCGATGTG	20	59.5
	qAv- IAG_R	AAATGCCAATTCATCTTCAGG	21	62.7
	qAv-RbL8_F	AACTGGAGATAGAGGCAAA	19	57.0
	qAv-RbL8_R	CCACCAGCAGCAATTC	16	59.4
	qTolC-F	GATTGGCGAAGATGCTGATG	20	61,7
	qTolC-R	ACTTCCATTCCCGCTGCT	18	60,8
	qVirB3_F	TGCAATGCTTTTTGGTGTGAG	21	62,1
	qVirB3_R	ATTTGGAGCATTTTCCCAATTTT	23	61,9
	qVirB8_F	TGATGCTCTTCAAGTGAGGTTTTC	24	62
	qVirB8_R	GATCATTCATTTCAAGCGATGC	22	61,8
	qwsp_F	TGGTGCAGCATTTACTCCAG	20	60,7
	qwsp_R	TCGCTTGATAAGCAAAACCA	20	57,7

Supplementary Table S1. Primers used to amplify the various sequences by qPCR or RT-qPCR. The primers were designed to amplify only the cDNA and do not amplify genomic DNA.
